# The effects of visual distractors on serial dependence

**DOI:** 10.1167/jov.23.12.1

**Published:** 2023-10-04

**Authors:** Christian Houborg, David Pascucci, Ömer Dağlar Tanrıkulu, Árni Kristjánsson

**Affiliations:** 1 Vision Sciences Laboratory, School of Health Sciences, University of Iceland, Reykjavik, Iceland; 2 Laboratory of Psychophysics, Ecole Polytechnique Fédérale de Lausanne (EPFL), Lausanne, Switzerland; 3 Vision Sciences Laboratory, School of Health Sciences, University of Iceland, Reykjavik, Iceland; 4 Department of Psychology, University of New Hampshire, Durham, NH, USA; 5 Vision Sciences Laboratory, School of Health Sciences, University of Iceland, Reykjavik, Iceland

**Keywords:** serial dependence, attentional suppression, spatial attention, distractor inhibition

## Abstract

Attractive serial dependence occurs when perceptual decisions are attracted toward previous stimuli. This effect is mediated by spatial attention and is most likely to occur when similar stimuli are attended at nearby locations. Attention, however, also involves the suppression of distracting information and of spatial locations where distracting stimuli have frequently appeared. Although distractors form an integral part of our visual experience, how they affect the processing of subsequent stimuli is unknown. Here, in two experiments, we tested serial dependence from distractor stimuli during an orientation adjustment task. We interleaved adjustment trials with a discrimination task requiring observers to ignore a peripheral distractor randomly appearing on half of the trials. Distractors were either similar to the adjustment probe (Experiment 1) or differed in spatial frequency and contrast (Experiment 2) and were shown at predictable or random locations in separate blocks. The results showed that the distractor caused considerable attentional capture in the discrimination task, with observers likely using proactive strategies to anticipate distractors at predictable locations. However, there was no evidence that the distractors affected the perceptual stream leading to positive serial dependence. Instead, they left a weak repulsive trace in Experiment 1 and more generally interfered with the effect of the previous adjustment probe in the serial dependence task. We suggest that this repulsive bias may reflect the operation of mechanisms involved in attentional suppression.

## Introduction

Our visual environment is a continuous stream. Information about objects and features constantly arrives at the retina in an overwhelming flow. The visual system therefore faces a major challenge in prioritizing information and tracking the history of task-relevant objects, while ignoring potential distractors and irrelevant details.

Recent research has shown that when we repeatedly attend to the same feature, such as the orientation or motion of a stimulus, our perceptual decisions become serially dependent: the stimulus features are judged as being more similar to recent past than they actually are (Fischer & Whitney, 2014; see Pascucci et al., 2023 for a review). This phenomenon has been reported in nearly all sorts of visual tasks and comes in many colors (Bliss, Sun, & D'Esposito, 2017; Ceylan et al., 2021; Collins, 2020; Collins, 2022; Fornaciai & Park, 2018b; Fritsche, Mostert, & de Lange, 2017; Fritsche & de Lange, 2019; Houborg, Kristjánsson, Tanrikulu & Pascucci, 2023; Manassi, Kristjánsson & Whitney, 2019; Murai & Whitney, 2021; Pascucci et al., 2019; Pascucci & Plomp, 2021a; Rafiei, Hansmann-Roth, Whitney, Kristjánsson, & Chetverikov, 2021a; Rafiei, Chetverikov, Hansmann-Roth, & Kristjánsson, 2021; Tanrikulu, Pascucci, & Kristjánsson, 2023). Such *attractive* serial dependence, in which decisions are biased *toward* prior stimuli, is considered to result from how the brain links together events across consecutive perceptual episodes (Corbett, Fischer, & Whitney, 2011; Fischer et al., 2020; Fischer & Whitney, 2014), a process that appears strongly influenced by attention (Fischer & Whitney, 2014; Pascucci et al., 2019). For instance, serial dependence is mostly evident when previous and current stimuli share a common feature that is attended to and they occur at nearby locations (Fischer & Whitney, 2014; Fritsche & de Lange, 2019). These aspects closely resemble the way attention has traditionally been considered to combine features into coherent objects, by binding together similar attended features, and only at similar attended locations (Kahneman, Treisman, & Gibbs, 1992; Treisman & Gelade, 1980).

Notably, however, research on this topic has typically manipulated attention by explicit instructions on what to attend to or not (Fischer & Whitney, 2014). Yet most of our everyday visual experience is made up of unexpected distracting stimuli that not only require no attention but also must be actively ignored to prevent interference with our current goals (Chelazzi, Marini, Pascucci, & Turatto, 2019). Whether and how the brain integrates such distractors in a sequence of perceptual episodes remains largely unknown.

### Attentional suppression

When we attend to an object, we rarely view that object in isolation. Visual scenes are cluttered with similar and distinct objects dispersed in space, which often surround and interfere with the current focus of our attention (Neisser, 1967; Tiurina, Markov, Choung, Herzog, & Pascucci, 2022; Treisman, 1988, see Kristjánsson & Egeth, 2020 for review). Sudden, unexpected stimuli can then abruptly capture our attention, breaking the current stream of perceptual and attentional focus (Chelazzi et al., 2019). Hence, our attentional system is constantly challenged to overcome the impact of irrelevant stimuli.

One way to efficiently overcome distraction is to anticipate the occurrence of irrelevant stimuli, for instance, by inferring their most likely features and locations from the statistics of visual events. This is evident in typical visual search tasks, where repeating the feature or location of a target leads to speeded and more accurate responses (Geng & Behrmann, 2002; Goschy, Bakos, Müller, & Zehetleitner, 2014; Maljkovic & Nakayama, 1994, Maljkovic & Nakayama, 1996; Pascucci & Turatto, 2013; van Moorselaar & Theeuwes, 2021, see Kristjánsson & Ásgeirsson, 2019; Ramgir & Lamy, 2022 for reviews). Priming phenomena of this kind are one of the most investigated examples, but there are more complex ways in which the brain may tune itself to avoid distraction. Recent research has clearly shown that when a distractor appears at predictable locations, these locations can be discounted from attentional focus and the distractor ceases to exert its original effect (Failing, Wang, & Theeuwes, 2019; Reder, Weber, Shang, & Vanyukov, 2003, see Gaspelin & Luck, 2019). Such learning of statistical regularities occurs regardless of awareness (Gao & Theeuwes, 2020, Gao & Theeuwes, 2022; Kristjánsson & Nakayama, 2003; Leber, Gwinn, Hong, & O'Toole, 2016) and typically leads to faster search times when a distractor is present at predictable locations or with predictable features. But the learning might also come at a cost, slowing search when a distractor is not present because observers “pre-activate” a resource-demanding attentional strategy against the expected distractor —that is, proactive filtering (Marini, Chelazzi, & Maravita, 2013; Marini, Demeter, Roberts, Chelazzi, & Woldorff, 2016; Wang & Theeuwes, 2018).

A still-debated topic concerns the underlying mechanisms and whether they involve, for instance, active suppression of distractor processing (Becker, Hemsteger, & Peltier, 2015; Cunningham & Egeth, 2016), faster reallocation of attention to the target (Chang, Cunningham, C., & Egeth, 2018), or the build-up of inhibitory templates that counteract distractor-related neural activity (Ramaswami, 2014). In this context, a recent line of work suggests that the ability to anticipate distractors may instantiate “rejection templates” that act as “negative” filters to suppress distractions (Arita, Carlisle, & Woodman, 2012; Beck & Hollingworth, 2015). Under this view, the action of a negative filter could result in negative serial dependence (i.e., a repulsive bias away from the features of a distractor encountered in the recent past).

In sum, the myriad of studies on distractor suppression suggests that when the properties of a distractor can be anticipated, the brain can successfully resist distraction and maintain focus on relevant aspects of visual input. It remains unclear, however, whether distractor stimuli leave any trace on the processing of subsequent task-relevant stimuli.

### Current aims

We sought to address a question that has not been considered before: does a distractor stimulus leave any discernible trace in a sequence of perceptual judgments? Previous studies have shown that attended stimuli cause serial dependence whereas unattended stimuli have no effect or even cause repulsive biases (Fischer & Whitney, 2014; Rafiei, Chetverikov, et al., 2021; Rafiei, Hansmann-Roth, et al., 2021). However, not attending to a stimulus is different from dealing with a distractor, and answering the question above can help to shed light on the neural fate of visual distractors and the mechanisms that facilitate their suppression.

**Figure 1.**
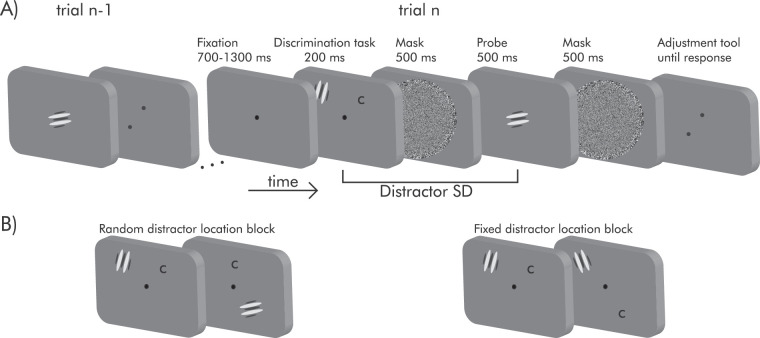
The paradigm in Experiments 1 and 2. (**A**) Example of a trial sequence: An orientation adjustment task was interleaved with a discrimination task. In the discrimination task, participants had to report whether the gap in a Landolt C was on the left or the right. On 50% of trials, a distractor (an oriented Gabor) was presented together with the target. Following a noise mask, the central Gabor of the adjustment task was presented, and participants had to reproduce its orientation by rotating a response tool. (**B**) In two different blocks, the distractor appeared either in a random location or at fully predictable locations (“random” and “fixed” conditions, respectively).

To this aim, we combined an orientation adjustment task with a secondary discrimination task (see Figure 1). In the discrimination task, observers focused on a Landolt C while a distractor stimulus appeared on 50% of trials. Crucially, the stimulus observers had to reproduce in the adjustment task (the probe), and the distractor in the discrimination task were both oriented Gabors. When these stimuli are attended, the orientation of the previous stimulus typically causes serial dependence in current decisions. Here, we made the previous Gabor a distractor and manipulated the probability of its location in two separate blocks. In the “random” block, the distractor could appear at one of four possible locations, randomly determined. In the “fixed” conditions, the distractor always appeared at the same location (see Methods). In the discrimination task, observers had to report the side of the gap in a Landolt C presented at one of the four locations, non-overlapping with the possible locations of the distractor. In two experiments, the distractor was exactly the same Gabor stimulus as the adjustment probe (Experiment 1) or differed in spatial frequency and contrast (Experiment 2). We measured serial dependence from the distractor as a function of its predictable or random location under the idea that predictable locations facilitate attentional mechanisms that anticipate, and therefore resist distractors more efficiently.

To investigate both the attractive and repulsive effects of the distractor on adjustment responses, we developed a novel method based on model selection, and evaluated models including attractive and repulsive components, as well as the effect of the predictability of the distractor. Furthermore, we considered serial dependence from the distractor, but also from the previously attended probe and its relation to the orientation of the distractor. Although we found no evidence for an effect of distractor predictability on neither the discrimination nor the adjustment task, our results indicate that distractors led to a weak but repulsive bias that affected current perceptual judgments and interfered with the trace of prior attended stimuli. In our paradigm, this effect appeared to be stronger when both the distractor and the probe were of high spatial frequency and low contrast (Experiment 1). We discuss these results in light of current theories of serial dependence and distractor processing, proposing that serial dependence can be a key to understanding the mechanisms of distractor suppression.

## General methods

### Ethics statement

The study was performed according to the requirements of the local ethics committee.

### Apparatus

The stimuli were generated with custom-made scripts from Matlab (2019b) and the Psychophysics Toolbox (Brainard, 1997) and presented on a 24-inch Asus monitor (resolution: 1920 × 1080 pixels, refresh rate: 60 Hz), on a Windows-based machine. The experiment was performed in a dimly lit room, and participants were positioned approximately 57 cm from the computer screen.

### Participants

Thirty-five healthy observers (age range 18–42, 15 females) from the University of Iceland community participated in the two experiments for a monetary reward (1500 ISK). Fifteen (one excluded, six female, age range 18–37) participated in Experiment 1, and 20 (two excluded, nine female, age range 22–42) in Experiment 2. The participants had normal or corrected-to-normal vision and were naïve about the purpose of the experiments. Written informed consent was collected from all participants beforehand.

### Stimuli and procedure

The experiments consisted of a series of trials where participants performed two sequential tasks (Figure 1). Each trial started with a fixation dot shown for 700 to 1300 ms. Participants then had to discriminate whether the gap in a Landolt C (peak contrast of 47%) was on the left or the right, while a distractor—a randomly oriented Gabor stimulus ranging from 0° to 180° in 20° steps—appeared on 50% of the trials, randomly determined. The distractor appeared at 8° off fixation, placed at the corners of an imaginary square. The Landolt C and distractor were presented for 200 ms and were followed by a noise mask for 500 ms (peak Michelson contrast = 75%, spatial frequency of 1 cpd, sigma of 2.75°). Participants had to report the side of the gap as quickly as possible by pressing the corresponding arrow key. After the discrimination task, a probe Gabor appeared at screen center, with a random orientation between 0° to 180° serving as the stimulus for the orientation adjustment task.

In Experiment 1 the probe and distractor Gabor stimuli were identical (i.e., same contrast and spatial frequency). In Experiment 2, both the contrast (peak Michelson = 10%) and the spatial frequency (0.25 cpd) of the probe Gabor were lower than those of the distractor stimuli. In both experiments, the adjustment stimuli had a random orientation difference with respect to the distractor, in the range between ±65° and were shown for 500 ms. The probe Gabor was followed by a noise mask, after which a response tool appeared at the center of the display. The response tool consisted of two dark-gray dots positioned at the end of an imaginary (nonvisible) line on a circle and participants had to rotate the line to reproduce the perceived orientation of the last Gabor. The initial orientation of the response tool was selected randomly. Participants could take as much time as they wanted and had to press the left mouse button to confirm their response, after which the next trial would proceed.

In both experiments, participants received verbal instructions and performed a sequence of practice trials under the supervision of the experimenter. Practice trials were not analyzed further. Participants were further instructed to ignore a distractor which appears in some trials, they were not informed about the locational probabilities in the blocked conditions. The two experiments were performed on two independent samples, and each experiment consisted of two separate sessions on separate days (one week was the maximum time allowed between sessions). Each session lasted approximately one hour and comprised of 1000 trials in total. During the experiments, participants were instructed to maintain their gaze on the fixation dot at screen center for the entire duration (breaks and between trials excluded). All stimuli were presented on the same gray background (83.33 cd/m^2^).

### Analysis

Before the main analyses, trials were removed in the following cases: 1) reaction times (RT) in the discrimination task outside the 200-1000 ms range; 2) adjustment errors detected as outliers in a two-step procedure, first excluding errors larger than ±45°, then excluding errors more than 1.5 interquartile ranges above the upper quartile or below the lower quartile; 3) adjustment errors slower than 10 seconds. By these exclusion criteria, 4.3% and 3.7% of the total trials were removed from Experiments 1 and 2, respectively. We also employed the following criteria for participant exclusion: 1) more than 25% of the trials were marked as outliers; 2) a circular correlation between the reported and presented orientation lower than 0.4; 3) accuracy and reaction times during the detection task three standard deviations away from the group mean. In Experiment 1, one participant was excluded for adding 90° to every response. In Experiment 2, one participant was excluded because of poor performance on the discrimination task (accuracy = 51%), and one participant exceeded the 25% of trials marked as outliers in the orientation adjustment task.

To analyze RT in the discrimination task, we used a general linear mixed-effects (GLM) regression (Matlab *fitglme*, distribution: inverse gaussian, link: identity), with RT as the dependent variable. The main predictors were the intercept plus the slope and the interaction associated with effect of the location condition (random vs. fixed, coded as binary variable) and distractor presence (absent vs. present, coded as a binary variable). In the GLM, we modelled the individual intercept as a random effect and excluded trials with incorrect discrimination responses.

For the serial dependence analyses, we used a novel approach. Serial dependence patterns typically contain a combination of repulsive and attractive effects due to prior stimuli (Pascucci et al., 2023). For example, several studies have shown attractive effects when prior stimuli are attended and reported, but repulsion, or a combination of the two otherwise (Pascucci et al., 2019; Pascucci & Plomp, 2021a). Because the Gabor stimulus in the discrimination task was a distractor, and therefore not attended, previous studies may therefore suggest a repulsive effect, or a combination of repulsion and attraction. However, the common approach of fitting the 1^st^ derivative of a Gaussian function (DoG; Fischer & Whitney, 2014) can only capture the effect that dominates (irrespective of whether it is repulsive or attractive). So, to provide a more exhaustive description of the bias, we developed a method based on model comparison.

Adjustment errors from all participants were aggregated into a pooled dataset and modelled with a set of linear regression models (see Table 1), including (1) a model with only an intercept (Δ0), (2) a model with an intercept and one variable accounting for the effect of the difference (Δ) between prior and current orientations (Δ1), (3) a model including a second variable accounting for an additional effect of Δ, which can model the combination of repulsive and attractive effects (Δ2), (4) a variant of Δ1 including the interaction between Δ and the random vs. fixed location condition (Δ1*loc), and (5) a variant of Δ2 including the interaction between both components related to Δ and the location condition (Δ2*loc). Since the effect of Δ on adjustment errors is typically nonlinear (i.e., it follows the shape of the DoG), the Δ variables used in each model were first transformed by multiplying Δ with a Gaussian function of variable width (from 10° to 80°) and choosing the best-fitting width (the one with the highest *r*^2^; Barbosa & Compte, 2020).

**Table 1.** Models of serial dependence in the analysis of experiments 1 and 2. Models are named as in Figures 2D and 4D. In all equations *y* is the adjustment error variable, *a* is the intercept, *b*_1,…, 6_ are the coefficients associated to each additional variable included in the model.

Model	Function
Δ0	*y* = *a*
Δ1	*y* = *a* + *b*Δ1
	*b*Δ1: difference between prior and current orientations
Δ2	*y* = *a* + *b*_1_Δ1 + *b*_2_Δ2
	*b*_1_Δ1, *b*_2_Δ2: attractive and repulsive effects
Δ1*loc	*y* = *a* + *b*_1_Δ1 + *b*_2_Δ1*b_3_*Loc*
	*b*_3_ *Loc*: dummy variable coding random vs. fixed location
Δ2*loc	*y* = *a* + *b*_1_Δ1 + *b*_2_Δ2 + *b*_3_Δ1*b_4_*Loc* + *b*_5_Δ2*b_6_*Loc*
	*b*_4/6_ *Loc*: dummy variable coding random vs. fixed location

We then performed model comparison using the Bayesian Information Criterion (BIC; Kass & Raftery, 1995; Raftery, 1995), selecting the model with the lowest BIC as the best. For further comparison we calculated the ΔBIC by subtracting the largest BIC from all the BIC values. ΔBIC can be considered as an approximation of the Bayes factor where a difference of 2 ΔBIC between models indicates positive evidence in support of the model with the lower BIC, and a value of 6 ΔBIC or above can be considered as strong positive evidence. This procedure was applied to analyze the errors as a function of both the distractor orientation on the immediately preceding discrimination task (distractor present trials) and the orientation of the last probe stimulus one trial before. Model comparison enabled the identification of effects caused by previous stimuli, their direction, the presence of multiple components of the opposite sign, and the interaction of these effects with the experimental conditions of interest while controlling for model complexity, thus providing a more exhaustive description of the data than the typical approach of fitting DoG functions.

## Results

### Experiment 1

Fourteen observers participated in Experiment 1, performing the discrimination task with a mean accuracy of 97% and mean RT of 498 ± 54.5 ms. The mean absolute error in the adjustment task was 9.29° ± 2.61° and response times were 1630 ± 310 ms.

**Figure 2.**
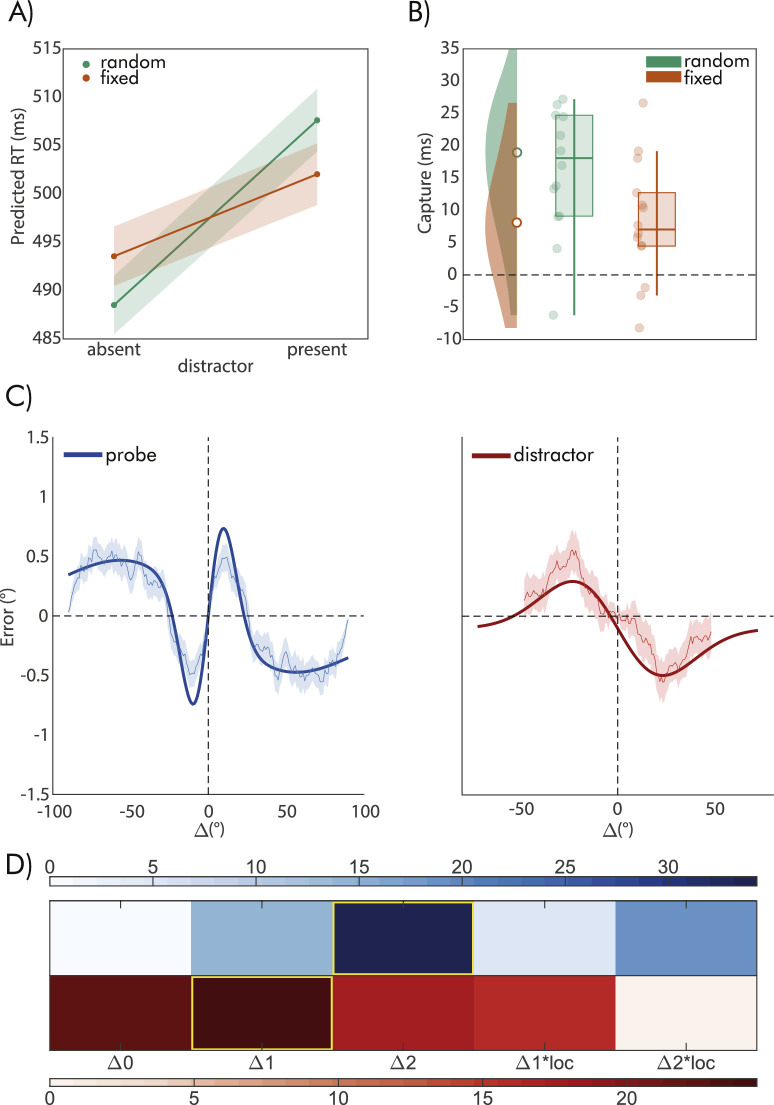
Results of Experiment 1: (**A**) Predicted RT mean and 95% CI from the GLM model estimating the effect of the distractor presence and location condition on RT. (**B**) Box plots, distributions, and individual datapoints of the capture effect on RT, computed as RT in distractor present minus absent trials, separately for the random (green items) and fixed (orange items) location conditions. (**C**, left plot) Errors (y axis) in the adjustment task as a function of the difference in orientation between the probe adjustment stimulus on the current and on the previous trial (Δ, x axis). (**C**, right plot) Errors as a function of the Δ between the current probe stimulus and the distractor in distractor-present trials. Data in C are moving averages of the group mean with 1 standard deviation smoothed in a 21° moving window, the fitted line is the predicted pattern from the best-fitting model (see Methods and Results). Models were fitted to the unsmoothed single-trial data. (**D**) Results from the model comparison applied to both the effect of prior probes (red color scale), and distractors (blue color scale). Values in the color scale reflect the difference in BIC values (ΔBIC) of each model from the one with the largest BIC (the one with the largest BIC has a value of 0, larger values indicate better fits, see Methods for the description of each model). The best-fitting models are highlighted in yellow.

For the discrimination task analyses, we modeled RT as a function of the presence/absence of a distractor and of the distractor location condition (fixed vs. random). The GLM results (see Methods) revealed a significant slope associated with the effect of a distractor, as well as with the effect of the distractor location and the interaction between the two (see Table 2). Observers were overall slower in the presence of a distractor, but the difference in RT varied as a function of whether the distractor location was fixed or random (Figure 2A). Although this pattern led to an apparent reduction of attentional capture (RT difference between distractor-present and distractor-absent trials) in the fixed location condition (Figure 2B), the interaction resulted from (1) a reduction of RT for distractor present trials in the fixed location condition and (2) an increase of RT for distractor absent trials in the fixed location condition (Figure 2A).

**Table 2.** GLM summary. Location refers to the random versus fixed location of the distractor (coded as a binary variable with random = 0). Distractor refers to the absence or presence of a distractor (coded as a binary variable with absent = 0). *Notes:* SE = standard error; DF, degree of freedom.

Variable	Estimate	SE	*t*-test	DF	*p* value	Lower	Upper
Intercept	489.29	13.93	35.13	13374	<0.001	461.99	516.59
Location	4.128	1.731	2.385	13374	0.0171	0.735	7.521
Distractor	18.53	1.786	10.38	13374	<0.001	15.029	22.03
Interaction	−10.97	2.517	−4.36	13374	<0.001	−15.90	−6.032

Together these results suggest that the fixed location condition adds a cost on distractor absent trials, which could be interpreted as a form of “proactive suppression” where observers expected and proactively filtered the distractor at the fixed location, leading to slower RT even when no distractor was present (Marini et al., 2013).

### Effect of distractor orientation on adjustment responses

After the discrimination task, observers performed an adjustment task on the same trial, with a new stimulus (probe) presented at the center. To examine the impact of the distractor orientation on the adjustment task, we used model comparison to assess various models predicting repulsive and attractive effects, as well as their interaction with fixed versus random distractor location conditions (see Methods). Our objective was to evaluate how the distractor influenced current perceptual decisions and whether this influence was affected by the predictability of the distractor location. The results of our RT analysis indicated that the distractor interfered with the discrimination task and that the predictability of its location also played a role, supporting the hypothesis that these effects may also manifest in serial dependence for the subsequent adjustment stimulus.

However, as revealed by the model comparison, although we found an overall effect of the distractor orientation, this effect was not modulated by the distractor location conditions. More precisely, when comparing models including no effect of the distractor (Δ0), a single effect (e.g., either attractive or repulsive, Δ1), a mixture of two effects (both attractive and repulsive, Δ1 + Δ2), and the interaction of these effects with the distractor location variable (Δ1 * loc, Δ2 * loc, see Figure 2, and Methods for details), the model with a single effect Δ1 was preferred over all the others (see Table 3). Model evidence was in favor of Δ1 against the model with the second smallest BIC (evidence in favor of Δ1 against Δ0, quantified by the ΔBIC = 2.20). Crucially, the effect fitted by the winning model was repulsive (i.e., a peak bias of −0.49°), indicating that observers were slightly biased away from the orientation of the distractor but independently of the location condition of the distractor. Similar repulsive effects of distractor stimuli have been reported in different contexts (Fischer & Whitney, 2014; Rafiei, Chetverikov, et al., 2021; Rafiei, Hansmann-Roth, et al., 2021).

**Table 3.** The set of models used in the serial dependence analysis and their associated BIC values for experiments 1 and 2. The results include models applied separately to the effect of the previous probe and the distractor. ΔBIC is reported for each model and computed as the difference between the model with the largest BIC (indicated as ΔBIC = 0) and all the other models. Larger ΔBIC values are indicative of better models.

	Distribution			
	Experiment 1		Experiment 2	
Model	Previous probe BIC	Distractor BIC	Previous probe BIC	Distractor BIC
Δ0	89322	44432	117487	58533
	ΔBIC = 0	ΔBIC = 22.30	ΔBIC = 0	ΔBIC = 29.38
Δ1	89305	44429	117364	58539
	ΔBIC = 14.77	ΔBIC = 24.50	ΔBIC = 117.31	ΔBIC = 23.39
Δ2	89286	44436	117336	58544
	ΔBIC = 34.29	ΔBIC = 17.46	ΔBIC = 151.46	ΔBIC = 17.58
Δ1 * loc	89314	44438	117374	58548
	ΔBIC = 5.50	ΔBIC = 15.79	ΔBIC = 107.96	ΔBIC = 14.74
Δ2 * loc	89300	44454	117355	58562
	ΔBIC = 19.27	ΔBIC = 0	ΔBIC = 132.56	ΔBIC = 0

### Effect of the previous probe on adjustment responses

To ensure that the observed repulsive effect of the distractor was indeed specific to the distractor, we conducted an analysis using the same model comparison as above, but with the aim of examining the impact of the previous probe (i.e., the stimulus presented on the preceding adjustment trial) on current adjustment errors. This served as a control to verify the presence of attractive serial dependence in our paradigm.

As expected, adjustment errors were systematically biased by the orientation of the previous probe, revealing a combination of attraction and repulsion, with the model predicting two components (Δ2) resulting in the best fit (see Table 3, Figure 4D). The best model (Δ2) was preferred over the second-best model, which included the distractor location effect (Δ2*loc, ΔBIC = 15.03). The attractive peak (0.76°) was evident for small Δ whereas the repulsive peak (−0.49°) occurred for larger Δ, as in typical patterns dominated by attractive serial dependence (Fritsche & de Lange, 2019).

### Interference between the distractor and the previous probe effect

**Figure 3.**
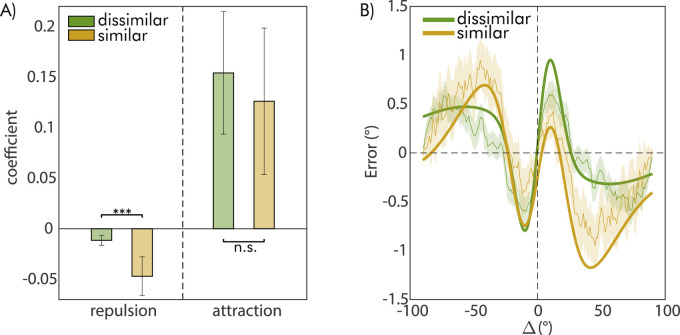
(**A**) Comparison of the effects of the previous probe as a function of whether the previous probe orientation was similar (within 45°, dark yellow bars) or different from the orientation of the distractor (above 45°, green bars). The two sets of bars refer to the attractive and repulsive components of the Δ2 model. Error bars are 95% CI, asterisks denote significant differences (*p = 0.05, **p = 0.01, ***p < 0.001). (B) Serial dependence curves and Δ2 model fit from the previous probe effect, represented with the same color coding as in A).

The results presented above suggest that adjustment errors were influenced by both the orientation of the distractor and the one of the previous probes, but in opposite ways. With additional analyses, we evaluated whether the two effects interacted with each other, specifically whether the impact of the previous probe changed depending on the similarity between its orientation and that of the distractor. If this were the case, it would suggest that the effect of prior stimuli, which are normally integrated with current ones, is diminished when prior stimuli are similar to irrelevant and distracting stimuli that are presented in between. To investigate this possibility, we divided the dataset into two halves based on the similarity between the previous probe and the distractor orientation (i.e., whether it was smaller or larger than 45°), and conducted a separate model comparison for the effect of the probe on each half. The model comparison results revealed no difference in the preferred model as a function of the similarity between the previous probe and the distractor, still favoring the Δ2 model with attractive and repulsive components (similar condition, ΔBIC = 14.5, dissimilar condition, ΔBIC = 18.80). For the condition in which the previous probe and distractor were dissimilar, the Δ2 was strongly favored over the second-best model (evidence in favor of Δ2 against Δ1, quantified by the ΔBIC = 10.38). However, for the condition where the probe and distractor orientation were similar, there was no conclusive evidence favoring the Δ2 model over the simplest Δ1 model (evidence in favor of Δ2 against Δ1, quantified by the ΔBIC = 0,74), suggesting that a single effect of Δ, characterized only by a repulsive component, provides a more parsimonious description of the data. In line with this, a direct comparison between the estimated coefficients of the two components from the Δ2 model, revealed that repulsion increased when the previous probe and the distractor had similar orientations (*z* = 3.51, *p* < 0.001, *z*-test for comparing coefficients between models, see Figure 3), whereas the attractive effect remained the same (*z* = 0.58, *p* = 0.56).

### Experiment 2

The serial dependence effects observed in Experiment 1, whether repulsive or attractive, were relatively small compared to previous studies (Fischer et al., 2020; Fritsche & de Lange, 2019; Kiyonaga, Scimeca, Bliss, & Whitney, 2017; Tanrikulu et al., 2023), with bias peaks of less than 1°. This could be because the high-spatial frequency stimuli used did not have enough uncertainty in their orientation content, an aspect that plays a key role in serial dependence (Ceylan, Herzog, & Pascucci, 2021; Cicchini, Mikellidou, & Burr, 2018; Tanrikulu et al., 2023; see review in Pascucci et al., 2023). It is therefore unclear whether the results of Experiment 1 can be applied to a broader context or if they may vary with modifications of the stimulus parameters.

In Experiment 2, the same paradigm was used, but with a reduction in contrast and spatial frequency of the probe Gabors. However, all other aspects of the experiment, including the contrast and spatial frequency of the distractor, remained the same as in Experiment 1. It is worth noting that the adjustment response bias caused by the distractor could potentially increase as a result of making the distractor more noticeable and less ambiguous than the probe.

Eighteen observers participated in this experiment, performing the discrimination task with a mean accuracy of 96% and a mean RT of 526 ± 86 ms. The mean absolute error in the adjustment task was 9.41° ± 2.10° and response times were 1600 ± 270 ms. The results of the GLM model on RT revealed a pattern in line with Experiment 1, showing a significant slope for the effect of distractor (absent vs. present), location (random vs. fixed), and their interaction (see Table 4).

**Table 4.** GLM model summary. The effects of distractor and location probability on performance in the discrimination task in experiment two. *Notes:* SE = standard error; DF, degree of freedom.

Variable	Estimate	SE	*t*-test	DF	*p* value	Lower	Upper
Intercept	517.35	19.44	26.61	16903	<0.001	479.24	555.45
Loc. Prob.	5.437	1.646	3.30	16903	<0.001	2.210	8.663
Distractor	15.30	1.687	9.07	16903	<0.001	11.99	18.60
Interaction	−7.241	2.388	−3.03	16903	0.002	−11.92	−2.561

### Effect of the distractor orientation on adjustment responses

**Figure 4.**
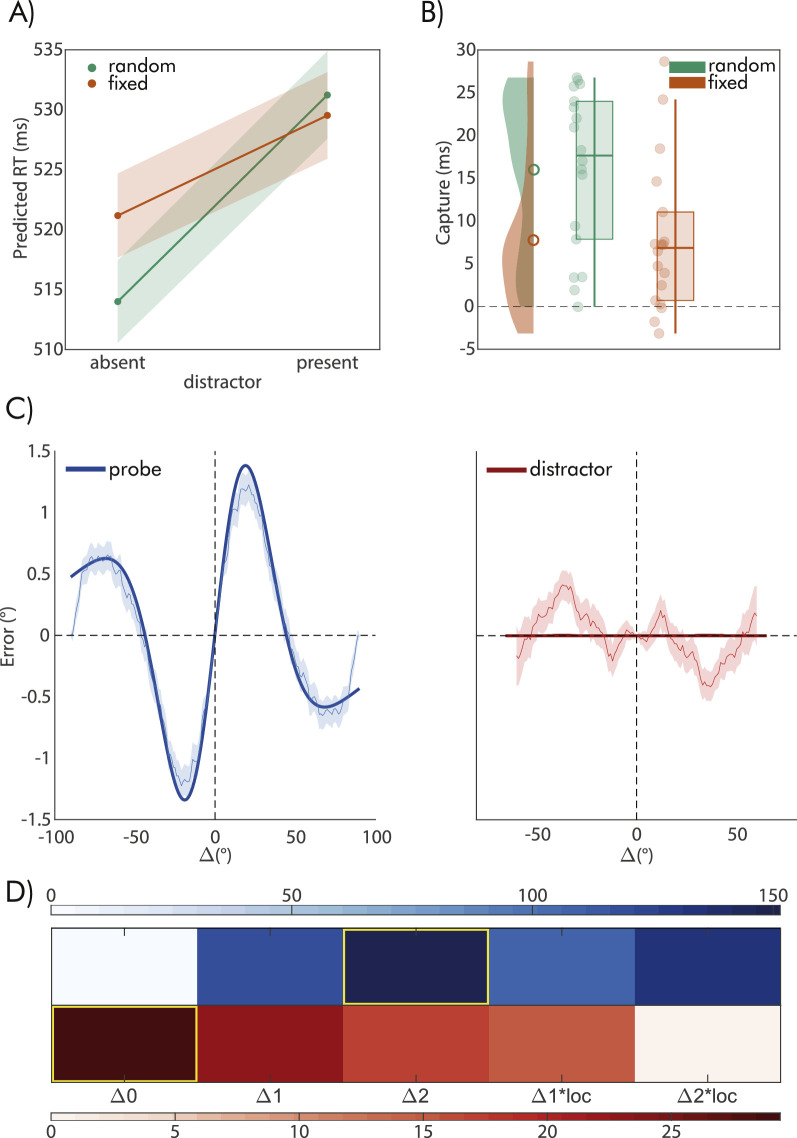
Results of Experiment 2: (**A**) Predicted RT mean and 95% CI from the GLM model on RT. (B) Capture effect on RT, computed as RT on distractor present minus absent trials, separately for the random (green items) and fixed (orange items) location conditions. (**C**, left plot) Errors (y-axis) on the adjustment task as a function of the difference in orientation between the probe adjustment stimulus on the current and the previous trial. (**C**, right plot) Errors as a function of the Δ between the current probe stimulus and the distractor in distractor-present trials. (**D**) Results from the model comparison applied to both the effect of prior probes (red color scale), and distractors (blue color scale). Values in the color scale are reported as in Figure 2. The best-fitting models are highlighted in yellow.

In contrast to the findings of the model comparison conducted in Experiment 1, the results of this experiment indicated no influence of the distractor on the adjustment task. All models that incorporated an effect of Δ performed worse than the null model, which predicted no impact of Δ (as shown in Figure 4C). Furthermore, the null model was considerably favored, even when compared to the simplest model with only one component, whether it be attractive or repulsive, linked to the effect of Δ (with a ΔBIC of 5.99, as displayed in Table 4).

### Effect of the previous probe on adjustment responses

When considering the effect of the previous probe, we observed a clear attractive bias, which was larger than in Experiment 1 (peak bias = 1.42°. Figure 4C). This trend, similar in shape and size to previous studies, was also accompanied by a repulsive component located at the tails (with a peak bias of −0.53°). The model comparison validated the existence of two components that accounted for the attractive and repulsive impact of Δ on adjustment errors, with evidence supporting the Δ2 model over the second-best fitting model (Δ2 * loc, with a ΔBIC of 18.90).

### Interference between the distractor and the previous probe effect

**Figure 5.**
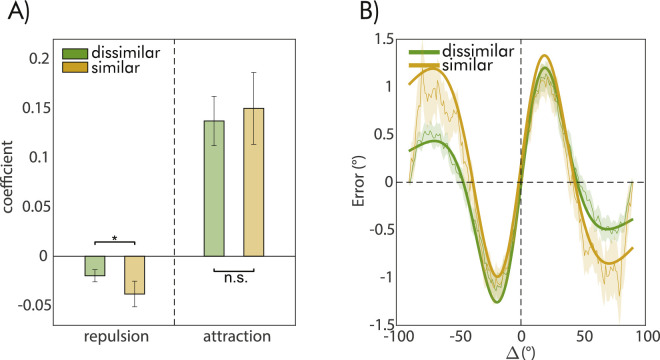
(**A**) Comparison of the effects of the previous probe as a function of whether the previous probe orientation was similar (within 45°, dark yellow bars) or different from the orientation of the distractor (above 45°, green bars) in Experiment 2. The two sets of bars refer to the attractive and repulsive components of the Δ2 model. Error bars are 95% CI, asterisks denote significant differences (*p = 0.05, **p = 0.01, ***p < 0.001). (B) Serial dependence curves and Δ2 model fit from the previous probe effect, represented with the same color coding as in A).

As in Experiment 1, we also performed a separate model comparison dividing the data into two halves based on the orientation similarity of the distractor and the previous probe (orientation difference smaller or larger than 45°). Model choice was not affected by dividing the data, and the preferred model remained the same in both conditions (e.g., Δ2), with strong evidence relative to the second-best models (evidence in favor of Δ2 against second choice, quantified by the ΔBIC > 10 in both conditions, see Figure 5B for the model fit).

However, a comparison of the coefficients accounting for repulsive and attractive effects as in Experiment 1, revealed a significant increase in the repulsive effect of the previous probe when its orientation was similar to the distractor (*z* = 2.53, *p* = 0.011, Figure 5), but no changes in the attractive effect (*z* = −0.57, *p* = 0.57, Figure 5). Hence, even though the probe effect always had both attractive and repulsive components, the strength of repulsion increased when a distractor with a similar orientation appeared right after the probe.

## General discussion

Our aim was to investigate the effects of visual distractors on serial dependence. While prior research suggests that serial dependence typically occurs when stimuli are attended, it remains unclear whether this effect extends to stimuli that capture attention but are ignored, or suppressed, as distractors. Additionally, we explored whether the predictability of the distractor location plays a role, as predictable distractors are typically easier for the visual system to ignore (Failing et al., 2019; van Moorselaar & Slagter, 2019; Wang & Theeuwes, 2018). To address these questions, we conducted two experiments that involved a discrimination task using Gabor stimuli as distractors, interleaved with an adjustment task involving task-relevant Gabor patches (probes). Our results show that visual distractors, exert a weak repulsive bias on subsequent decisions and interfere with the attractive serial dependence typically induced by preceding attended and task-relevant stimuli.

Previous studies do not provide a conclusive understanding of how the visual system integrates prior and current information when the preceding history includes both relevant and irrelevant stimuli. Some studies conclude that serial dependence occurs independently of the task and that the task relevance of a stimulus does not play a role (Fornaciai & Park, 2018a; Murai & Whitney, 2021). In contrast, other studies have shown repulsive effects due to stimuli that were not attended to or not reported in the recent past (Fischer & Whitney, 2014; Fornaciai & Park, 2018b; Pascucci et al., 2019; Pascucci & Plomp, 2021b; Rafiei, Chetverikov, et al., 2021; Rafiei, Hansmann-Roth, et al., 2021). However, none of these studies have examined the effect of an "irrelevant" stimulus that the visual system must suppress to prevent distraction. Here, we found that distractors are not integrated in the perceptual stream that leads to attractive serial dependence, even when they are highly similar to other stimuli in close temporal and spatial proximity. This finding is particularly relevant because distractor stimuli can capture attention and, therefore, they might be processed to some degree (Moher & Egeth, 2012), which differs from prior studies where irrelevant stimuli were simply ignored, as there was no accompanying task.

### The repulsive serial dependence from distractor stimuli

In the two experiments, the repulsive effect of visual distractors was either weak (Experiment 1) or absent (Experiment 2). This could have two explanations. First, the different results may be due to between-experiment variability. Second, the modified parameters could have been the critical factor. In Experiment 2, we used more uncertain stimuli as probes in the adjustment task (e.g., Gabor of lower contrast and spatial frequencies). As attractive serial dependence increases under uncertainty (Ceylan et al., 2021; Cicchini et al., 2018; Tanrikulu et al., 2023), this might have led to an overall dominance of attractive biases due to the previous probe and a reduced measurable effect of repulsion caused by the distractor. Recent studies have suggested that attractive and repulsive components of serial dependence are additive and arise concurrently, so that in the absence of one, the other will be more dominant (Fornaciai & Park, 2019; Moon & Kwon, 2022; Pascucci et al., 2019; Sheehan & Serences, 2022). Under this view, it is plausible that repulsive effects from distractors are only seen when all the stimuli are highly reliable and any attractive bias towards prior stimuli is overall reduced. By this logic, we speculate that the lower spatial frequency of the probe in Experiment 2, which is typically associated with more uncertain stimuli (Ceylan et al., 2021; Cicchini et al., 2018; Tanrikulu et al. 2023), might have fostered attractive influences of the previous probe orientation that outweighed any potential repulsive effect of the distractor. Nevertheless, the distractor orientation interfered with the effect of the previous probe in both experiments. In particular, the repulsive effect of the previous probe, evident for large orientation differences between consecutive probes, was further increased when the previous probe was similar to the distractor, indicating an indirect effect that still supports a repulsive component related to distractor processing, which shaped the effect of previous probes.

### The effect of statistical regularities on attentional suppression

Our results also speak to potential effects of manipulating the predictability of distractor locations. Specifically, RTs in the discrimination task, were reduced when the distractor appeared at a fixed and predictable location compared to a random location. However, we also observed an increase in RT in blocks where the distractor was absent, but its location was predictable. This pattern, which was consistent across both experiments, suggests that manipulating the distractor location not only affected the capture caused by the distractor, but also led to an overall change in attentional strategy. One explanation is that participants proactively expected the distractor at a fixed location and engaged anticipatory filtering mechanisms, which come at the cost of an overall increase in RT (Marini et al., 2013). It is worth highlighting that our manipulation of presenting the distractor at a fixed location for entire blocks differed from the more implicit manipulations of statistical regularities often employed in visual attention research (e.g., Leber et al., 2016), and this difference might have prompted different attentional strategies in our study. Nonetheless, our findings add to a large body of literature indicating that the attentional system is sensitive to both explicit and implicit regularities in distractor events, allowing the system to build a model of what is irrelevant and distracting, and when and where it is likely to occur (Pascucci & Turatto, 2015; Turatto, Bonetti, & Pascucci, 2018; Turatto & Pascucci, 2016).

A central question is what implications the internal model has, especially with regard to developing a model of what should be ignored, such as a distractor (Ramaswami, 2014; Turatto, Bonetti, Pascucci, & Chelazzi, 2018). There is open debate that focuses on whether the attentional system would benefit from so-called “negative models” or “rejection templates,” which, rather than facilitating specific target features, would help to prevent allocating attention to nontarget elements (Carlisle & Kristjánsson, 2018; Woodman, Carlisle, & Reinhart, 2013; see Chelazzi et al., 2019 for a review). For example, it has been proposed that negative models may involve inhibitory mechanisms in sensory processing (Ramaswami, 2014), whereas other studies argue that evidence of rejection templates is only the indirect consequence of building models of what is relevant and attended (Ferrante et al., 2018; Sauter, Liesefeld, Zehetleitner, & Müller, 2018). Our study demonstrates that knowing the location of a distractor in advance can speed up performance in the relevant discrimination task but also has a weak repulsive effect on subsequent stimuli and interferes with the effect of previous stimuli. This suggests that the occurrence of distractors leaves a negative (repulsive) trace that persists for a few seconds, in line with the notion of sensory suppression mechanisms (Ramaswami, 2014). Another debated issue is whether ignoring a distractor involves initial processing of the distractor and faster reallocation of attention away from it, or no processing at all (Cunningham & Egeth, 2016; Ferrante, Zhigalov, Hickey, & Jensen, 2023; Moher & Egeth, 2012). Our results seem to suggest that distractors, at least in our paradigm, were processed to the extent that they left a trace or interfered with other ongoing traces of prior stimuli.

Our findings show that the predictability of the distractor location did not affect serial dependence in either of the experiments. Therefore it is difficult to draw conclusions about the impact of more efficient suppression resulting from statistical regularities. Instead, the observed effects are most likely a consequence of the distractor's capture rather than its suppression, which was strong and evident in both the predictable and unpredictable location conditions. Despite this, this finding is important because it shows that distractors that capture attention are not incorporated into sequential perceptual decisions the way relevant stimuli are. Thus, the results reported here add to research showing that perceptual decisions are biased away from the features of recent distractors (Rafiei, Chetverikov, et al., 2021; Rafiei, Hansmann-Roth, et al., 2021). In a broader context, the rationale behind our study may provide a basis for future research exploiting serial dependence as a tool to understand the mechanisms implicated in distractor processing and the neural outcomes of such stimuli, by measuring the imprint left by a distractor on perceptual processing.

## Conclusions

Our aim was to shed light on the role of attention in serial dependence, by revealing the effects of attentional distractors, when they can or cannot be suppressed through location suppression. We found no evidence of positive serial dependence caused by distractor stimuli but some evidence of negative serial dependence. This result may provide a starting point for future research elucidating the perceptual consequences of attentional filtering and the fate of successfully suppressed stimulus features in the context of spatiotemporal continuity in vision.
